# A novel approach based on neutrosophic Bonferroni mean operator of trapezoidal and triangular neutrosophic interval environments in multi-attribute group decision making

**DOI:** 10.1038/s41598-023-37497-z

**Published:** 2023-06-28

**Authors:** D. Nagarajan, A. Kanchana, Kavikumar Jacob, Nasreen Kausar, Seyyed Ahmad Edalatpanah, Mohd Asif Shah

**Affiliations:** 1 Department of Mathematics, Rajalakshmi Institute of Technology, Kuthambakkam, Chennai, India; 2 Department of Mathematics and Statistics, Fuzzy Mathematics and Application Group, Faculty of Applied Sciences and Technology, Universiti Tun Hussein Onn Malaysia, Pagoh Campus, 84600 Pagoh, Johor Malaysia; 3 Department of Mathematics, Faculty of Arts and Science, Yildiz Technical University, Esenler, 34220 Istanbul, Turkey; 4 Department of Applied Mathematics, Ayandegan Institute of Higher Education, Tonekabon, Iran; 5 Department of Economics, College of Business and Economics, Kebri Dehar University, PO Box 250, Kebri Dehar, Ethiopia; 6 School of Business, Woxsen University, Kamkole, Sadasivpet, Hyderabad, 502345 Telangana India; 7 Division of Research and Development, Lovely Professional University, Phagwara, 144001 Punjab India

**Keywords:** Mathematics and computing, Applied mathematics, Computational science, Pure mathematics

## Abstract

Neutrosophic multicriteria is a method of decision-making that uses indeterminacy to combine several criteria or elements, frequently with incomplete or ambiguous information, to find a solution. The neutrosophic multicriteria analysis enables the assessment of qualitative and subjective aspects and can assist in resolving conflicting goals and preferences. In the Neutrosophic Multi-Attribute Group Decision Making (NMAGDM) problems, all the information provided by the decision makers (DMs) is expressed as single value neutrosophic triangular and trapezoidal numbers examined in this study which can provide more flexibility and accuracy in capturing uncertainty and aggregating preferences. We offer a novel approach for determining the neutrosophic possibility degree of two and three trapezoidal and triangular neutrosophic sets and the concepts of neutrosophic possibility mean value. The trapezoidal and triangular neutrosophic Bonferroni mean (TITRNBM) operator and the trapezoidal and triangular neutrosophic weighted Bonferroni mean (TITRNWBM) operator are two aggregation methods we then create. Further, we examine the TITRNBM and TITRNWBM attributes and their uniqueness. The NMAGDM approach with trapezoidal and triangular information is suggested based on the TITRNWBM operator and possibility degree. Finally, a concrete example of manufacturing companies searching for the best supplier for assembling the critical parts is provided to validate the established strategies and show their practical applicability and efficacy.

## Introduction

The problem of group decision-making in multi-attributes using Fuzzy (FMAGDM) is the method used to find the feasible alternative when the group decision makers' information is uncertain because of the complication in the social environment, economic environment, and unclear subject thinking of human nature. In past years several methods were used to solve the above problem (FMAGDM) with the help of type-1 fuzzy sets. Chen^1^ used the extension of the TOPSIS method. Chen^2^ explained the evaluation of aggregative risk rate utilizing the development of software of fuzzy sets in the fuzzy group decision-making environment. Wang and Lin^3^ developed a method of selecting the configuration items using the software. The compromise programming method solves the above problem (FMAGDM) by taking the aggregating function as Li^4^ introduces fuzzy weighted Minkowski distance. A maximizing deviation method is used to solve the above problem (FMAGDM) in the linguistic atmosphere by Wu and Chen^5^. The practical, interactive procedure used for solving the above method (FMAGDM), where the attribute weights are given partially, is explained by Xu^6^. Lin and Wu^7^ use a simple analytical method. Also, a computing coordination method was proposed to solve the above method (FMAGDM) was explained by Tsai and Wang^8^. A multi-granularity linguistic method is used to solve the above problem (FMAGDM) is explained in Fan and Liu^9^. Using the non-homogenous information, different distance values have been measured, and a different method is developed to solve the above (FMAGDM) presented in Li^10^. However, all the above method is based on type-1 fuzzy sets.

Zadeh^11^ first initiated the extension of type-1 fuzzy sets to type-2 fuzzy sets. The type-2 fuzzy set solved more uncertainty compared to the type-1 fuzzy set by crisp the numbers of the type-1 fuzzy set as used as the membership value for type-2 fuzzy sets introduced in Wu and Mendel^12^. Because of the complexity of using type-2 fuzzy sets, the interval type-2 fuzzy sets were presented by Mendal et al.^13^ and applied in many practical applications, as given in Mendal and Wu^14–17^. A new Linguistic weighted average method is imposed in interval type-2 fuzzy sets to solve the above problem (FMAGDM) given in Wu and Mendal^18,19^. The FMAGDM is solved using the ranking method and arithmetic operations in interval type-2 fuzzy sets, as provided by Chen and Lee^20^. Also, the TOPSIS method is applied to solve. FMAGDM uses interval type-2 fuzzy sets, which is explained in Chen and Lee^21^. Even though the attribute weights are partially known, the interval type-2 fuzzy set used in characterizing the attribute values is clearly explained in Wang and Liu^22^. A ranking method in interval type-2 fuzzy set to solve the FMAGDM method is introduced by Chen and Yang^23^. A new method was introduced to solve FMAGDM using a trapezoidal interval type-2 fuzzy set by Zhang and Zhang^24^. A technique of possibility degree used to solve the FMAGDM problem is explained in Hu et al.^25^.

The Bonferroni operator is a mathematical operator used in decision-making and statistical analysis. Its advantages make it a valuable tool in specific contexts, such as conservatism, simplicity, broad applicability, flexibility and control of Type I errors. FMAGDM models can capture and handle the uncertainty and ambiguity inherent in decision-making processes by employing the neutrosophic Bonferroni operator. The Neutrosophic Bonferroni operator considers each attribute's membership, indeterminacy, and non-membership degrees, providing a comprehensive and balanced aggregation of the decision-maker's opinions. The scope of the neutrosophic Bonferroni operator in FMAGDM lies in its ability to handle uncertain and indeterminate information. It enables decision-makers to express their preferences more nuancedly, leading to more accurate and reliable decision outcomes. However, it is essential to note that the neutrosophic Bonferroni operator is just one of many methods available for FMAGDM, and its suitability depends on the specific context and requirements of the decision problem at hand. A Bonferroni^26^ mean operator is used in interval type-2 fuzzy set to solve the FMAGDM problem, as explained in Gong et al.^27^. Using the weighted geometric Bonferroni operator and the possible degree of interval type-2 fuzzy set, an FMAGDM problem is solved. Peng and Smarandache^28^ examined the single-valued neutrosophic Dombi Bonferroni mean operator, single-valued neutrosophic weighted Dombi Bonferroni mean operator, single-valued neutrosophic Dombi geometric Bonferroni mean operator and single-valued neutrosophic weighted Dombi geometric Bonferroni mean operator mobile to illustrate the cloud computing industry decision making problem. Awang et al.^29^ proposed an interval-valued neutrosophic Shapley normalized weighted Bonferroni mean (INSNWBM) operator and explored the applicability of the INSNWBM in decision-making. Banerjee et al.^30^ introduced the generalized partitioned Bonferroni mean (GPBM) in an interval-valued fuzzy set based on the strict t-conorms additive generators. They developed the interval valued GPBM (IVGPBM) and the weighted IVGPBM (WIVGPBM) aggregation operators to study multi-attribute decision-making problems. Deli^31^ developed the generalized trapezoidal hesitant fuzzy Bonferroni arithmetic mean operator and generalized trapezoidal hesitant fuzzy Bonferroni geometric mean operator for aggregating the generalized trapezoidal hesitant fuzzy information in order to discuss the multi-attributes decision-making problems. Further, the multi-attribute group decision-making problems analysed by Bonferroni mean operators in the picture hesitant fuzzy numbers environments^32^. Ali et al.^33^ derived complex q-rung orthopair fuzzy Archimedean Bonferroni mean and weighted Bonferroni mean operators and examined the interrelationship among the finite number of attributes in modern data fusion theory. Chakraborty and Saha^34^ studied the Fermatean fuzzy multi-criteria group decision making with the help of Bonferroni mean and weighted Bonferroni mean operators for the healthcare industry. In order to improve the possibility degree and a better solution in this paper, we use the single value neutrosophic method. The accuracy has been increased when using the neutrosophic rather than the interval type-2 fuzzy sets and other generalizations. Hence, we present a new method to solve the FMAGDM problem by using two aggregate methods, such as the trapezoidal and triangular neutrosophic Bonferroni mean (TITRNBM) operator and the trapezoidal and triangular neutrosophic weighted Bonferroni mean (TITRNWBM) operator. FMAGDM problem is to find the most desirable alternative from a set of feasible alternatives, where the information provided by a group of decision makers is usually uncertain or fuzzy due to the increasing complexity of the socio-economic environment and the vagueness of inherent subjective nature of human thinking. Hence, the significant advantage of the proposed operator is that they have considered the interrelationships of aggregated values. Hence, we have employed an illustration related to a manufacturing company searching for the best supplier for assembling the critical parts to validate the proposed operators' practical applicability and effectiveness.

Thus, the paper is formulated in “Introduction” section, where the basic definitions and operations of single value neutrosophic are given. In “Preliminaries” section, the neutrosophic possibility mean has been introduced for both the lower and upper mean. Here a new method is presented for deriving the above mean values. In “Single value neutrosophic possibility mean value and possibility degree” section, the triangular single value neutrosophic operator and weighted mean have been found. In “Neutrosophic Bonferroni operators” section, The trapezoidal single value neutrosophic Bonferroni operator and weighted mean have been found with some related properties, and different approaching cases are also discussed. “Proposed method” section discusses steps for solving the FMAGDM problem using a trapezoidal single value neutrosophic operator and weighted mean. In “Illustrative example” section, the above steps were imposed with the help of an illustrative example, and a comparison with the previous results is given. Finally, in “Conclusion”, the conclusion is given.

## Preliminaries

In this section, we define some definitions and arithmetic operations for a neutrosophic set used in forthcoming sections.

### **Definition 1**

(*Smarandache*^35^) Let $U$ be the universal set, then neutrosophic set is defined as $A=\{\left({T}_{A}\left(x\right), {I}_{A}\left(x\right),{F}_{A}\left(x\right)\right),x\in U\}$ where ${T}_{A}\left(x\right), {I}_{A}\left(x\right),{F}_{A}\left(x\right)\in ]{0}^{-},{1}^{+}[$ and ${{0}^{-}\le T}_{A}\left(x\right)+{I}_{A}\left(x\right)+{F}_{A}\left(x\right)\le {3}^{+}$.

### **Definition 2**

(*Wang*^36^) Let $U$ be the universal set, then SVNS is defined as $\dot{A}=\{\left({T}_{\dot{A}}\left(x\right), {I}_{\dot{A}}\left(x\right),{F}_{\dot{A}}\left(x\right)\right),x\in U\}$ where ${T}_{\dot{A}}\left(x\right), {I}_{\dot{A}}\left(x\right),{F}_{\dot{A}}\left(x\right)\in [\mathrm{0,1}]$ and ${0\le T}_{\dot{A}}\left(x\right)+{I}_{\dot{A}}\left(x\right)+{F}_{\dot{A}}\left(x\right)\le 3$.

The proposed definition of this paper are defined as follows,

### **Definition 3**

The proposed score function for the neutrosophic triangular set is given by,

1 $${\dot{S}}^{*}\left({T}_{A}\left(x\right), {I}_{A}\left(x\right),{F}_{A}\left(x\right)\right)=\frac{1}{2}\left({1+T}_{A}\left(x\right)-2*{I}_{A}\left(x\right)+{F}_{A}\left(x\right)\right)$$

### **Definition 4**

The proposed score function for the neutrosophic trapezoidal set is given by,

2 $${\dot{\ddot{S}}}^{*}\left({T}_{A}\left(x\right), {I}_{A}\left(x\right),{F}_{A}\left(x\right)\right)=\frac{1}{2}\left({1+n*T}_{A}\left(x\right)-{I}_{A}\left(x\right)+{F}_{A}\left(x\right)\right)$$

where $n$ represents number of terms in the matrices.

### Single value neutrosophic possibility mean value and possibility degree

Here, the single value neutrosophic possibility mean value and the single value neutrosophic possibility degree are explained. In the beginning, the lower and upper neutrosophic possibility mean, are defined as follows.

#### **Definition 5**

If a single value neutrosophic number is

$$\left(TN,IN,FN\right)=\left(\left(T{N}^{U},I{N}^{U},F{N}^{U}\right),\left(T{N}^{L},I{N}^{L},F{N}^{L}\right)\right),$$

where $\left(T{N}^{U},I{N}^{U},F{N}^{U}\right)$ and $\left(T{N}^{L},I{N}^{L},F{N}^{L}\right)$ are the upper and lower neutrosophic member function having the level set as

$$\left(T{N}_{\alpha }^{U},I{N}_{\alpha }^{U},F{N}_{\alpha }^{U}\right)=\left[\left(T{N}_{1}^{U}\left(\alpha \right),I{N}_{1}^{U}\left(\alpha \right),{FN}_{1}^{U}\left(\alpha \right)\right),\left({TN}_{2}^{U}\left(\alpha \right),{IN}_{2}^{U}\left(\alpha \right),F{N}_{2}^{U}\left(\alpha \right)\right)\right], \alpha \in \left[\left(\mathrm{0,0},0\right),\left(T{h}_{U},I{h}_{U},F{h}_{U}\right)\right]$$ and $$\left(T{N}_{\beta }^{L},I{N}_{\beta }^{L},F{N}_{\beta }^{L}\right)=\left[\left(T{N}_{1}^{L}\left(\beta \right),I{N}_{1}^{L}\left(\beta \right),{FN}_{1}^{L}\left(\beta \right)\right),\left(T{N}_{2}^{L}\left(\beta \right),{IN}_{2}^{L}\left(\beta \right),{FN}_{2}^{L}\left(\beta \right)\right)\right],\alpha \in \left[\left(\mathrm{0,0},0\right),\left(T{h}_{L},I{h}_{L},F{h}_{L}\right)\right]$$

where $\left(T{h}_{U},I{h}_{U},F{h}_{U}\right)$ and $\left(T{h}_{L},I{h}_{L},F{h}_{L}\right)$ are the highest and lower membership neutrosophic function of ${N}^{U}$ and ${N}^{L}$.

#### **Definition 6**

The lower neutrosophic possibility mean for $N=\left({N}^{U},{N}^{L}\right)$ is given by,

3 $$ \begin{aligned}\left(T{\widetilde{M}}_{*}\left(N\right),I{\widetilde{M}}_{*}\left(N\right),F{\widetilde{M}}_{*}\left(N\right)\right)&=\left({\int }_{0}^{T{h}_{U}}{\left(T{N}_{1}^{U}\left(\alpha \right)\right)}^{\alpha }d\alpha +{\int }_{0}^{T{h}_{L}}{\left(T{N}_{1}^{L}\left(\beta \right)\right)}^{\beta }d\beta ,{\int }_{0}^{I{h}_{U}}{\left(I{N}_{1}^{U}\left(\alpha \right)\right)}^{\alpha }d\alpha\right. \\ & \quad \left.+{\int }_{0}^{I{h}_{L}}{\left(I{N}_{1}^{L}\left(\beta \right)\right)}^{\beta }d\beta ,{\int }_{0}^{F{h}_{U}}{\left(F{N}_{1}^{U}\left(\alpha \right)\right)}^{\alpha }d\alpha +{\int }_{0}^{F{h}_{L}}{\left(F{N}_{1}^{L}\left(\beta \right)\right)}^{\beta }d\beta \right)\end{aligned} $$

where $\left(T{\widetilde{M}}_{*}\left(N\right),I{\widetilde{M}}_{*}\left(N\right),F{\widetilde{M}}_{*}\left(N\right)\right)$ is the arithmetic mean of members of the neutrosophic membership function.

#### **Definition 7**

The upper neutrosophic possibility mean for $\left(TN,IN,FN\right)=\left(\left(T{N}^{U},I{N}^{U},F{N}^{U}\right),\left(T{N}^{L},I{N}^{L},F{N}^{L}\right)\right)$ is given by,

4 $$ \begin{aligned}\left({T\widetilde{M}}^{*}\left(N\right),I{\widetilde{M}}^{*}\left(N\right),F{\widetilde{M}}^{*}\left(N\right)\right)&=\left({\int }_{0}^{T{h}_{U}}{\left(T{N}_{2}^{U}\left(\alpha \right)\right)}^{\alpha }d\alpha +{\int }_{0}^{T{h}_{L}}{\left(T{N}_{2}^{L}\left(\beta \right)\right)}^{\beta }d\beta ,{\int }_{0}^{I{h}_{U}}{\left(I{N}_{2}^{U}\left(\alpha \right)\right)}^{\alpha }d\alpha \right. \\ & \quad \left. +{\int }_{0}^{I{h}_{L}}{\left(I{N}_{2}^{L}\left(\beta \right)\right)}^{\beta }d\beta ,{\int }_{0}^{F{h}_{U}}{\left(F{N}_{2}^{U}\left(\alpha \right)\right)}^{\alpha }d\alpha +{\int }_{0}^{F{h}_{L}}{\left(F{N}_{2}^{L}\left(\beta \right)\right)}^{\beta }d\beta \right)\end{aligned} $$

where $\left({T\widetilde{M}}^{*}\left(N\right),I{\widetilde{M}}^{*}\left(N\right),F{\widetilde{M}}^{*}\left(N\right)\right)$ is the arithmetic mean of members of the neutrosophic membership function.

#### **Definition 8**

The notation gives the closed bounded interval of the neutrosophic lower and upper mean value $\left(T\widetilde{M}\left(N\right),I\widetilde{M}\left(N\right),F \widetilde{M}\left(N\right)\right)=\left[\left(T{\widetilde{M}}_{*}\left(N\right),I{\widetilde{M}}_{*}\left(N\right),F{\widetilde{M}}_{*}\left(N\right)\right),\left({T\widetilde{M}}^{*}\left(N\right),I{\widetilde{M}}^{*}\left(N\right),F{\widetilde{M}}^{*}\left(N\right)\right)\right]$.

#### **Definition 9**

Similarly, the neutrosophic mean value of $\left(T{N}_{1},I{N}_{1},F{N}_{1}\right)$ and $\left(T{N}_{2},I{N}_{2},F{N}_{2}\right)$ is given by,

$$\left(T\widetilde{M}\left({N}_{1}\right),I\widetilde{M}\left({N}_{1}\right),F \widetilde{M}\left({N}_{1}\right)\right)=\left[\left(T{\widetilde{M}}_{*}\left({N}_{1}\right),I{\widetilde{M}}_{*}\left({N}_{1}\right),F{\widetilde{M}}_{*}\left({N}_{1}\right)\right),\left({T\widetilde{M}}^{*}\left({N}_{1}\right),I{\widetilde{M}}^{*}\left({N}_{1}\right),F{\widetilde{M}}^{*}\left({N}_{1}\right)\right)\right]$$ and 

$$\left(T\widetilde{M}\left({N}_{2}\right),I\widetilde{M}\left({N}_{2}\right),F \widetilde{M}\left({N}_{2}\right)\right)=\left[\left(T{\widetilde{M}}_{*}\left({N}_{2}\right),I{\widetilde{M}}_{*}\left({N}_{2}\right),F{\widetilde{M}}_{*}\left({N}_{2}\right)\right),\left({T\widetilde{M}}^{*}\left({N}_{2}\right),I{\widetilde{M}}^{*}\left({N}_{2}\right),F{\widetilde{M}}^{*}\left({N}_{2}\right)\right)\right]$$.

#### **Definition 10**

The possibility neutrosophic degree is given as $\left(p\left({TN}_{1}\succcurlyeq {TN}_{2}\right),p\left({IN}_{1}\succcurlyeq {IN}_{2}\right),p\left(F{N}_{1}\succcurlyeq {FN}_{2}\right)\right)$

5 $$=\left(\begin{array}{c}\mathrm{min}\left\{\mathrm{max}\left(\frac{T{\widetilde{M}}^{*}\left({N}_{1}\right)-T{\widetilde{M}}_{*}\left({N}_{2}\right)}{{T\widetilde{M}}^{*}\left({N}_{1}\right)-T{\widetilde{M}}_{*}\left({N}_{1}\right)+{T\widetilde{M}}^{*}\left({N}_{2}\right)-T{\widetilde{M}}_{*}\left({N}_{2}\right)},0\right),1\right\},\mathrm{min}\left\{\mathrm{max}\left(\frac{I{\widetilde{M}}^{*}\left({N}_{1}\right)-I{\widetilde{M}}_{*}\left({N}_{2}\right)}{{\widetilde{IM}}^{*}\left({N}_{1}\right)-{I\widetilde{M}}_{*}\left({N}_{1}\right)+{I\widetilde{M}}^{*}\left({N}_{2}\right)-{I\widetilde{M}}_{*}\left({N}_{2}\right)},0\right),1\right\},\\ \mathrm{min}\left\{\mathrm{max}\left(\frac{F{\widetilde{M}}^{*}\left({N}_{1}\right)-F{\widetilde{M}}_{*}\left({N}_{2}\right)}{{F\widetilde{M}}^{*}\left({N}_{1}\right)-F{\widetilde{M}}_{*}\left({N}_{1}\right)+{F\widetilde{M}}^{*}\left({N}_{2}\right)-F{\widetilde{M}}_{*}\left({N}_{2}\right)},0\right),1\right\}\end{array}\right)$$

#### **Definition 11**

The possibility neutrosophic degree $\left(p\left({TN}_{1}\succcurlyeq {TN}_{2}\right),p\left({IN}_{1}\succcurlyeq I{N}_{2}\right),p\left({FN}_{1}\succcurlyeq {FN}_{2}\right)\right)$ has to satisfy the following property.

i. $$(\mathrm{0,0},0)\le \left(p\left({TN}_{1}\succcurlyeq {TN}_{2}\right),p\left({IN}_{1}\succcurlyeq I{N}_{2}\right),p\left({FN}_{1}\succcurlyeq {FN}_{2}\right)\right)\le (\mathrm{1,1},1)$$ and $$\left(\mathrm{0,0},0\right)\le \left(p\left({TN}_{2}\succcurlyeq T{N}_{1}\right),p\left({IN}_{2}\succcurlyeq I{N}_{1}\right),p\left({FN}_{2}\succcurlyeq F{N}_{1}\right)\right)\le \left(\mathrm{1,1},1\right).$$
ii. If $\left(T{\widetilde{M}}_{*}\left({N}_{1}\right),I{\widetilde{M}}_{*}\left({N}_{1}\right),F{\widetilde{M}}_{*}\left({N}_{1}\right)\right)=\left(T{\widetilde{M}}_{*}\left({N}_{2}\right),I{\widetilde{M}}_{*}\left({N}_{2}\right),F{\widetilde{M}}_{*}\left({N}_{2}\right)\right)$ and $\left({T\widetilde{M}}^{*}\left({N}_{1}\right),I{\widetilde{M}}^{*}\left({N}_{1}\right),F{\widetilde{M}}^{*}\left({N}_{1}\right)\right)=\left({T\widetilde{M}}^{*}\left({N}_{2}\right),I{\widetilde{M}}^{*}\left({N}_{2}\right),F{\widetilde{M}}^{*}\left({N}_{2}\right)\right)$, then $\left(p\left(T{N}_{1}\succcurlyeq {TN}_{1}\right),p\left({IN}_{1}\succcurlyeq I{N}_{1}\right),p\left({FN}_{1}\succcurlyeq F{N}_{1}\right)\right)=\left(\mathrm{0.5,0.5,0.5}\right)$.
iii. For a neutrosophic member $\left({TN}_{1},{IN}_{1},F{N}_{1}\right),\left(T{N}_{2},I{N}_{2},F{N}_{2}\right),\left(T{N}_{3},I{N}_{3},F{N}_{3}\right)$, if $\left(p\left({TN}_{1}\succcurlyeq {TN}_{2}\right),p\left({IN}_{1}\succcurlyeq I{N}_{2}\right),p\left({FN}_{1}\succcurlyeq {FN}_{2}\right)\right)=(\mathrm{0.5,0.5,0.5})$ and $\left(p\left(T{N}_{2}\succcurlyeq {TN}_{3}\right),p\left({IN}_{2}\succcurlyeq {IN}_{3}\right),p\left(F{N}_{2}\succcurlyeq F{N}_{3}\right)\right)=(\mathrm{0.5,0.5,0.5})$ then $\left(p\left({TN}_{1}\succcurlyeq {TN}_{3}\right),p\left(I{N}_{1}\succcurlyeq {IN}_{3}\right),p\left(F{N}_{1}\succcurlyeq F{N}_{3}\right)\right)=(\mathrm{0.5,0.5,0.5})$.
iv. For a neutrosophic member $\left({TN}_{1},{IN}_{1},F{N}_{1}\right),\left(T{N}_{2},I{N}_{2},F{N}_{2}\right),\left(T{N}_{3},I{N}_{3},F{N}_{3}\right)$, if $\left(p\left({TN}_{1}\succcurlyeq {TN}_{2}\right),p\left({IN}_{1}\succcurlyeq I{N}_{2}\right),p\left({FN}_{1}\succcurlyeq {FN}_{2}\right)\right)=(\mathrm{0.5,0.5,0.5})$ and $\left(p\left(T{N}_{2}\succcurlyeq {TN}_{3}\right),p\left({IN}_{2}\succcurlyeq {IN}_{3}\right),p\left(F{N}_{2}\succcurlyeq F{N}_{3}\right)\right)=(\mathrm{0.5,0.5,0.5})$ then $\left(p\left({TN}_{1}\succcurlyeq {TN}_{2}\right),p\left({IN}_{1}\succcurlyeq I{N}_{2}\right),p\left({FN}_{1}\succcurlyeq {FN}_{2}\right)\right)+\left(p\left(T{N}_{2}\succcurlyeq {TN}_{3}\right),p\left({IN}_{2}\succcurlyeq {IN}_{3}\right),p\left(F{N}_{2}\succcurlyeq F{N}_{3}\right)\right)=2\left(p\left({TN}_{1}\succcurlyeq {TN}_{3}\right),p\left(I{N}_{1}\succcurlyeq {IN}_{3}\right),p\left(F{N}_{1}\succcurlyeq F{N}_{3}\right)\right)$.

#### **Definition 12**

For the neutrosophic trapezoidal number $\left(TN,IN,FN\right)=\left(\left(T{N}^{U},I{N}^{U},F{N}^{U}\right),\left(T{N}^{L},I{N}^{L},F{N}^{L}\right)\right)=\left(\left(\left(T{a}_{1}^{U},I{a}_{1}^{U},F{a}_{1}^{U}\right),\left(T{a}_{2}^{U},I{a}_{2}^{U},F{a}_{2}^{U}\right),\left(T{a}_{3}^{U},I{a}_{3}^{U},F{a}_{3}^{U}\right),\left(T{a}_{4}^{U},I{a}_{4}^{U},F{a}_{4}^{U}\right),\left(T{h}^{U},I{h}^{U},F{h}^{U}\right)\right)\right.$, $\left.\left(\left(T{a}_{1}^{L},I{a}_{1}^{L},F{a}_{1}^{L}\right), \left(T{a}_{2}^{L},I{a}_{2}^{L},F{a}_{2}^{L}\right), \left(T{a}_{3}^{L},I{a}_{3}^{L},F{a}_{3}^{L}\right),\left(T{a}_{4}^{L},I{a}_{4}^{L},F{a}_{4}^{L}\right),\left(T{h}^{L},I{h}^{L},F{h}^{L}\right)\right)\right)$, the lower neutrosophic possibility mean is calculated by,

6 $$\left(T{\widetilde{M}}_{*}\left(N\right),I{\widetilde{M}}_{*}\left(N\right),F{\widetilde{M}}_{*}\left(N\right)\right)=\left(\begin{array}{c}{\int }_{0}^{T{h}_{U}}{\left(T{a}_{1}^{U}+\frac{T{a}_{2}^{U}-T{a}_{1}^{U}}{T{h}_{U}}\right)}^{\alpha }d\alpha +{\int }_{0}^{T{h}_{L}}{\left(T{a}_{1}^{L}+\frac{T{a}_{2}^{L}-T{a}_{1}^{L}}{T{h}_{L}}\right)}^{\beta }d\beta ,\\ {\int }_{0}^{I{h}_{U}}{\left(I{a}_{1}^{U}+\frac{I{a}_{2}^{U}-I{a}_{1}^{U}}{I{h}_{U}}\right)}^{\alpha }d\alpha +{\int }_{0}^{I{h}_{L}}{\left(I{a}_{1}^{L}+\frac{I{a}_{2}^{L}-I{a}_{1}^{L}}{I{h}_{L}}\right)}^{\beta }d\beta ,\\ {\int }_{0}^{F{h}_{U}}{\left(F{a}_{1}^{U}+\frac{F{a}_{2}^{U}-F{a}_{1}^{U}}{F{h}_{U}}\right)}^{\alpha }d\alpha +{\int }_{0}^{F{h}_{L}}{\left(F{a}_{1}^{L}+\frac{F{a}_{2}^{L}-F{a}_{1}^{L}}{F{h}_{L}}\right)}^{\beta }d\beta ,\end{array}\right)$$

7 $$ \begin{aligned}&=\left(\left(\frac{1}{6}\left(T{a}_{1}^{U}+2T{a}_{2}^{U}\right)T{h}_{U}^{2}+\frac{1}{6}\left(T{a}_{1}^{L}+2T{a}_{2}^{L}\right)T{h}_{L}^{2}\right),\left(\frac{1}{6}\left(I{a}_{1}^{U}+2I{a}_{2}^{U}\right){Ih}_{U}^{2}+\frac{1}{6}\left(I{a}_{1}^{L}+2I{a}_{2}^{L}\right)I{h}_{L}^{2}\right),\right.\\ & \quad \left. \left(\frac{1}{6}\left(F{a}_{1}^{U}+2F{a}_{2}^{U}\right)F{h}_{U}^{2}+\frac{1}{6}\left(F{a}_{1}^{L}+2F{a}_{2}^{L}\right)F{h}_{L}^{2}\right)\right)\end{aligned} $$

#### **Definition 13**

For the neutrosophic trapezoidal number $\left(TN,IN,FN\right)=\left(\left(T{N}^{U},I{N}^{U},F{N}^{U}\right),\left(T{N}^{L},I{N}^{L},F{N}^{L}\right)\right)=\left(\left(\left(T{a}_{1}^{U},I{a}_{1}^{U},F{a}_{1}^{U}\right),\left(T{a}_{2}^{U},I{a}_{2}^{U},F{a}_{2}^{U}\right),\left(T{a}_{3}^{U},I{a}_{3}^{U},F{a}_{3}^{U}\right),\left(T{a}_{4}^{U},I{a}_{4}^{U},F{a}_{4}^{U}\right),\left(T{h}^{U},I{h}^{U},F{h}^{U}\right)\right)\right.,$ $\left.\left(\left(T{a}_{1}^{L},I{a}_{1}^{L},F{a}_{1}^{L}\right),\left(T{a}_{2}^{L},I{a}_{2}^{L},F{a}_{2}^{L}\right),\left(T{a}_{3}^{L},I{a}_{3}^{L},F{a}_{3}^{L}\right),\left(T{a}_{4}^{L},I{a}_{4}^{L},F{a}_{4}^{L}\right),\left(T{h}^{L},I{h}^{L},F{h}^{L}\right)\right)\right),$ the upper neutrosophic possibility mean is calculated by,

8 $$\left({T\widetilde{M}}^{*}\left(N\right),I{\widetilde{M}}^{*}\left(N\right),F{\widetilde{M}}^{*}\left(N\right)\right)=\left(\begin{array}{c}{\int }_{0}^{T{h}_{U}}{\left(T{a}_{4}^{U}+\frac{T{a}_{3}^{U}-T{a}_{4}^{U}}{T{h}_{U}}\right)}^{\alpha }d\alpha +{\int }_{0}^{T{h}_{L}}{\left(T{a}_{4}^{L}+\frac{T{a}_{3}^{L}-T{a}_{4}^{L}}{T{h}_{L}}\right)}^{\beta }d\beta ,\\ {\int }_{0}^{I{h}_{U}}{\left(I{a}_{4}^{U}+\frac{I{a}_{3}^{U}-I{a}_{4}^{U}}{I{h}_{U}}\right)}^{\alpha }d\alpha +{\int }_{0}^{I{h}_{L}}{\left(I{a}_{4}^{L}+\frac{I{a}_{3}^{L}-I{a}_{4}^{L}}{I{h}_{L}}\right)}^{\beta }d\beta ,\\ {\int }_{0}^{F{h}_{U}}{\left(F{a}_{4}^{U}+\frac{F{a}_{3}^{U}-F{a}_{4}^{U}}{F{h}_{U}}\right)}^{\alpha }d\alpha +{\int }_{0}^{F{h}_{L}}{\left(F{a}_{4}^{L}+\frac{F{a}_{3}^{L}-F{a}_{4}^{L}}{F{h}_{L}}\right)}^{\beta }d\beta ,\end{array}\right)$$

9 $$ \begin{aligned} & = \left(\left(\frac{1}{6}\left(T{a}_{4}^{U}+2T{a}_{3}^{U}\right)T{h}_{U}^{2}+\frac{1}{6}\left(T{a}_{4}^{L}+2T{a}_{3}^{L}\right)T{h}_{L}^{2}\right),\left(\frac{1}{6}\left(I{a}_{4}^{U}+2I{a}_{3}^{U}\right){Ih}_{U}^{2}+\frac{1}{6}\left(I{a}_{4}^{L}+2I{a}_{3}^{L}\right)I{h}_{L}^{2}\right),\right.\\ & \quad \left. \left(\frac{1}{6}\left(F{a}_{4}^{U}+2F{a}_{3}^{U}\right)F{h}_{U}^{2}+\frac{1}{6}\left(F{a}_{4}^{L}+2F{a}_{3}^{L}\right)F{h}_{L}^{2}\right)\right)\end{aligned} $$

#### **Definition 14**

The neutrosophic preference matrix $\left(TP,IP,FP\right)$ is given as

$$\left(TP,IP,FP\right)=\left(\begin{array}{c}\begin{array}{cc}\left(p\left(T{N}_{1}\succcurlyeq {TN}_{1}\right),p\left({IN}_{1}\succcurlyeq I{N}_{1}\right),p\left({FN}_{1}\succcurlyeq F{N}_{1}\right)\right)& \left(p\left({TN}_{1}\succcurlyeq {TN}_{2}\right),p\left({IN}_{1}\succcurlyeq I{N}_{2}\right),p\left({FN}_{1}\succcurlyeq {FN}_{2}\right)\right)\\ \left(p\left({TN}_{2}\succcurlyeq {TN}_{1}\right),p\left({IN}_{2}\succcurlyeq I{N}_{1}\right),p\left({FN}_{2}\succcurlyeq {FN}_{1}\right)\right)& \left(p\left({TN}_{2}\succcurlyeq {TN}_{2}\right),p\left({IN}_{2}\succcurlyeq I{N}_{2}\right),p\left({FN}_{2}\succcurlyeq {FN}_{2}\right)\right)\end{array}\\ \begin{array}{cc}\vdots & \vdots \\ \left(p\left({TN}_{n}\succcurlyeq {TN}_{1}\right),p\left({IN}_{n}\succcurlyeq I{N}_{1}\right),p\left({FN}_{n}\succcurlyeq {FN}_{1}\right)\right)& \left(p\left({TN}_{n}\succcurlyeq {TN}_{2}\right),p\left({IN}_{n}\succcurlyeq I{N}_{2}\right),p\left({FN}_{n}\succcurlyeq {FN}_{2}\right)\right)\end{array}\end{array}\right.$$

10 $$\left.\begin{array}{c}\begin{array}{cc}\cdots & \left(p\left({TN}_{1}\succcurlyeq {TN}_{n}\right),p\left({IN}_{1}\succcurlyeq I{N}_{n}\right),p\left({FN}_{1}\succcurlyeq {FN}_{n}\right)\right)\\ \cdots & \left(p\left({TN}_{1}\succcurlyeq {TN}_{n}\right),p\left({IN}_{1}\succcurlyeq I{N}_{n}\right),p\left({FN}_{1}\succcurlyeq {FN}_{n}\right)\right)\end{array}\\ \begin{array}{cc}\vdots & \vdots \\ \cdots & \left(p\left({TN}_{n}\succcurlyeq {TN}_{n}\right),p\left({IN}_{n}\succcurlyeq I{N}_{n}\right),p\left({FN}_{n}\succcurlyeq {FN}_{n}\right)\right)\end{array}\end{array}\right)$$

#### **Definition 15**

The neutrosophic ranking value $\mathcal{R}\left(TN,IN,FN\right)$ is given by,

11 $$ \begin{aligned}\mathcal{R}\left(TN,IN,FN\right)&=\left(\frac{1}{n(n-1)}\left({\oplus }_{k=1}^{n}p\left(T{N}_{1}\succcurlyeq {TN}_{k}\right)+\frac{n}{2}-1\right),\frac{1}{n(n-1)}\left({\oplus }_{k=1}^{n}p\left({IN}_{1}\succcurlyeq I{N}_{k}\right)+\frac{n}{2}-1\right),\right.\\&\quad\left.\frac{1}{n(n-1)}\left({\oplus }_{k=1}^{n}p\left({FN}_{1}\succcurlyeq F{N}_{k}\right)+\frac{n}{2}-1\right)\right)\end{aligned} $$

where $\oplus$ is an additive operator.

### Neutrosophic Bonferroni operators

Bonferroni^37^ first introduced the aggregation operator for mean. Yagar^38^ was generalized with the help of the OWA operator and the Choquet integral. Beliakov^39^ also gives a reformed generalized above method. In Gong et al.^27^, the interval type-2 Bonferroni mean operator is defined. This can be extended in this paper; a proposed neutrosophic Bonferroni operator is defined, and its aggregating operations will be defined.

#### **Definition 16**

Let

$$ \begin{aligned}& \left(T{N}_{i},I{N}_{i},F{N}_{i}\right)=\left(\left(T{N}_{i}^{U},I{N}_{i}^{U},F{N}_{i}^{U}\right),\left(T{N}_{i}^{L},I{N}_{i}^{L},F{N}_{i}^{L}\right)\right)\\ &=\left(\begin{array}{c}\left(\left(T{a}_{i1}^{U},I{a}_{i1}^{U},F{a}_{i1}^{U}\right),\left(T{a}_{i2}^{U},I{a}_{i2}^{U},F{a}_{i2}^{U}\right),\left(T{a}_{i3}^{U},I{a}_{i3}^{U},F{a}_{i3}^{U}\right),\left(T{a}_{i4}^{U},I{a}_{i4}^{U},F{a}_{i4}^{U}\right),\left(T{h}_{i}^{U},I{h}_{i}^{U},F{h}_{i}^{U}\right)\right),\\ \left(\left(T{a}_{i1}^{L},I{a}_{i1}^{L},F{a}_{i1}^{L}\right),\left(T{a}_{i2}^{L},I{a}_{i2}^{L},F{a}_{i2}^{L}\right),\left(T{a}_{i3}^{L},I{a}_{i3}^{L},F{a}_{i3}^{L}\right),\left(T{a}_{i4}^{L},I{a}_{i4}^{L},F{a}_{i4}^{L}\right),\left(T{h}_{i}^{L},I{h}_{i}^{L},F{h}_{i}^{L}\right)\right)\end{array}\right)\end{aligned} $$

where $(i=\mathrm{1,2},\dots ,n)$ be the set of neutrosophic member and for $s,t\ge 0$, we define neutrosophic Bonferroni mean as

12 $$ \begin{aligned}& {NBM}^{\left(s,t\right)}\left(\left(T{N}_{1},I{N}_{1},F{N}_{1}\right),\left(T{N}_{2},I{N}_{2},F{N}_{2}\right),\dots ,\left(T{N}_{n},I{N}_{n},F{N}_{n}\right)\right)\\ &=\left(\frac{1}{s+t}{\left({\otimes }_{\begin{array}{c}i,j=1\\ i\ne j\end{array}}^{n}\left(sT{N}_{i}\oplus tT{N}_{j}\right)\right)}^{\frac{1}{n(n-1)}},\frac{1}{s+t}{\left({\otimes }_{\begin{array}{c}i,j=1\\ i\ne j\end{array}}^{n}\left(sI{N}_{i}\oplus tI{N}_{j}\right)\right)}^{\frac{1}{n(n-1)}},\right.\\&\quad\left.\frac{1}{s+t}{\left({\otimes }_{\begin{array}{c}i,j=1\\ i\ne j\end{array}}^{n}\left(sF{N}_{i}\oplus tF{N}_{j}\right)\right)}^{\frac{1}{n(n-1)}}\right)\end{aligned} $$

where $\otimes$ is a multiplicative operator.

The proposed neutrosophic Bonferroni operator has the enormous advantage of high flexibility with adjustable parameters. The neutrosophic Bonferroni operator combines the membership values and indeterminacy values of two neutrosophic sets to determine the membership and indeterminacy of the resulting set. To some extent, the neutrosophic Bonferroni operator aims to preserve the indeterminacy of the input sets. The operator tends to propagate or retain the indeterminacy in the resulting set by taking the maximum indeterminacy values. This property distinguishes it from other operators that may not explicitly address indeterminacy preservation.

## Proposed method

In order to handle the problem of group decision making of multi attributes in the environment of single value neutrosophic trapezoidal and triangular member. We start the problem with $Y$ alternatives with G attributes where $Y=\left\{{y}_{1},{y}_{2},\dots ,{y}_{n}\right\}$ and G = $\left\{{g}_{1},{g}_{2},\dots ,{g}_{m}\right\}$. Now we assume that there are $l$ number of decision makers say ${D}_{1},{D}_{2},\dots ,{D}_{l}.$ Let $\left(T{\mathcal{R}}^{(k)},I{\mathcal{R}}^{(k)},F{\mathcal{R}}^{(k)}\right)={\left({TA}_{ij}^{(k)},{IA}_{ij}^{(k)},{FA}_{ij}^{(k)}\right)}_{nXm}$ be an single value neutrosophic decision matrix, where $\left({TA}_{ij}^{(k)},{IA}_{ij}^{(k)},{FA}_{ij}^{(k)}\right)$ is a neutrosophic member given by the decision makers ${D}_{i}$ to the alternatives ${y}_{j}$ whose corresponding attributes is ${g}_{k}$. Two types of attributes depend on benefits and cost. The attributes are divided into two sets of attributes and depend on benefit and cost, respectively. Also, it satisfies the condition ${G}_{1}\cup {G}_{2}=G$ and ${G}_{1}\cap {G}_{2}=\varnothing$. After that normalization should be done for $\left(T{\mathcal{R}}^{(k)},I{\mathcal{R}}^{(k)},F{\mathcal{R}}^{(k)}\right)$ depends on the attributes ${g}_{k}\left(k=\mathrm{1,2},\dots ,m\right)$. In this paper we start with the given neutrosophic member

13 $$\left(T{\mathcal{R}}^{\left(k\right)},I{\mathcal{R}}^{\left(k\right)},F{\mathcal{R}}^{\left(k\right)}\right)=\left({TA}_{ij}^{\left(k\right)},{IA}_{ij}^{\left(k\right)},{FA}_{ij}^{\left(k\right)}\right)=\left\{\begin{array}{l}\left({TA}_{ij}^{\left(k\right)},{IA}_{ij}^{\left(k\right)},{FA}_{ij}^{\left(k\right)}\right), if j\in {G}_{1}\\ \left({\left({TA}_{ij}^{\left(k\right)}\right)}^{c},{\left({IA}_{ij}^{\left(k\right)}\right)}^{c},{\left({FA}_{ij}^{\left(k\right)}\right)}^{c}\right), if j\in {G}_{2}\end{array}\right.$$

where $\left({\left({TA}_{ij}^{(k)}\right)}^{c},{\left({IA}_{ij}^{(k)}\right)}^{c},{\left({FA}_{ij}^{(k)}\right)}^{c}\right)$ is the complement value of $\left({TA}_{ij}^{(k)},{IA}_{ij}^{(k)},{FA}_{ij}^{(k)}\right)$. Thus, we finally reached the normalized neutrosophic decision matrix $\left(T{\mathcal{R}}^{(k)},I{\mathcal{R}}^{(k)},F{\mathcal{R}}^{(k)}\right)={\left({TA}_{ij}^{(k)},{IA}_{ij}^{(k)},{FA}_{ij}^{(k)}\right)}_{nXm}$. In this problem, the final opinion is obtained by considering individual decisions in the group decision. We use the additive and scalar multiplicative operation to convert the normalized neutrosophic individual decision matrix $\left(T{\mathcal{R}}^{(k)},I{\mathcal{R}}^{(k)},F{\mathcal{R}}^{(k)}\right)={\left({TA}_{ij}^{(k)},{IA}_{ij}^{(k)},{FA}_{ij}^{(k)}\right)}_{nXm}$ into the joint normalized neutrosophic collective decision matrix given as $\left(T\mathcal{R},I\mathcal{R},F\mathcal{R}\right)={\left(T{A}_{ij},{IA}_{ij},F{A}_{ij}\right)}_{nXm}$ where $\left(T{A}_{ij},{IA}_{ij},F{A}_{ij}\right)=\left(\sum_{k=1}^{l}T{\lambda }_{k}{A}_{ij}^{(k)},\sum_{k=1}^{l}I{\lambda }_{k}{A}_{ij}^{(k)},\sum_{k=1}^{l}F{\lambda }_{k}{A}_{ij}^{(k)}\right)$.If the weight of the attribute is clearly known, that is, the neutrosophic weight vector $\left(Tw,Iw,Fw\right)={\left(T{w}_{i},I{w}_{i},F{w}_{i}\right)}_{lXm}$ of the attributes $\left(T{g}_{j},I{g}_{j},F{g}_{j}\right)$ which can be obtained in advance. Then we use the neutrosophic Bonferroni mean to solve the group decision making of multi attributes, which can be given in Algorithm 1.

**Algorithm 1:**

**Table:** 

Step 1: Convert the given problem attributes into a neutrosophic trapezoidal or triangular member.
Step 2: With the help of neutrosophic collective decision matrix $\left(T\mathcal{R},I\mathcal{R},F\mathcal{R}\right)={\left(T{A}_{ij},{IA}_{ij},F{A}_{ij}\right)}_{nXm}$ and the neutrosophic weight vector $\left(Tw,Iw,Fw\right)={\left(T{w}_{i},I{w}_{i},F{w}_{i}\right)}_{lXm}$ The neutrosophic weighted Bonferroni mean is given by $$\left(T{d}_{k},I{d}_{k},F{d}_{k}\right)={NWBM}_{w}^{(s,t)}\left(\left(T{N}_{k,1},I{N}_{k,1},F{N}_{k,1}\right),\left(T{N}_{k,2},I{N}_{k,2},F{N}_{k,2}\right),\dots ,\left(T{N}_{k,n},I{N}_{k,n},F{N}_{k,m}\right)\right)=\left(\frac{1}{s+t}{\left({\otimes }_{\begin{array}{c}i,j=1\\ i\ne j\end{array}}^{m}\left(s{\left(T{N}_{ki}\right)}^{{w}_{i}}\oplus t{\left(T{N}_{kj}\right)}^{{w}_{j}}\right)\right)}^{\frac{1}{m(m-1)}},\frac{1}{s+t}{\left({\otimes }_{\begin{array}{c}i,j=1\\ i\ne j\end{array}}^{m}\left(s{\left(I{N}_{ki}\right)}^{{w}_{i}}\oplus t{\left(I{N}_{kj}\right)}^{{w}_{j}}\right)\right)}^{\frac{1}{m(m-1)}}, \frac{1}{s+t}{\left({\otimes }_{\begin{array}{c}i,j=1\\ i\ne j\end{array}}^{m}\left(s{\left(F{N}_{ki}\right)}^{{w}_{i}}\oplus t{\left(F{N}_{kj}\right)}^{{w}_{j}}\right)\right)}^{\frac{1}{m(m-1)}}\right) \quad \quad \quad (14)$$ where $\left(k=\mathrm{1,2},\dots ,n\right)$ and $s=t=1$.
Step 3: Use additive and multiplicative operation to determine the neutrosophic lower and upper mean value is given as $\left(T\widetilde{M}\left({d}_{k}\right),I\widetilde{M}\left({d}_{k}\right),F \widetilde{M}\left({d}_{k}\right)\right)$
Step 4: Use scalar multiplicative to determine the value of possibility neutrosophic degree, $\left(p\left({TN}_{i}\succcurlyeq {TN}_{j}\right),p\left({IN}_{i}\succcurlyeq {IN}_{j}\right),p\left(F{N}_{i}\succcurlyeq {FN}_{j}\right)\right), i=\mathrm{1,2},3 \;\; and \;\; j=\mathrm{1,2},3$.
Step 5: Then find the neutrosophic preference matrix $\left(TP,IP,FP\right)$ using (10).
Step 6: Find the neutrosophic ranking value $\mathcal{R}\left(T{N}_{i},I{N}_{i},F{N}_{i}\right)$ using (11).
Step 7: Finally using (2), the rank of the alternatives $\mathcal{R}({N}_{i})$ is obtained.
Step 8: The ranking of alternatives will be given in the descending order.
Step 9: Comparison with the previous results.

## Illustrative example

Step 1: Consider the problem given in Gong et al.^27^; first, the given problem will be fully defined as a neutrosophic trapezoidal or triangular member. In this section, we give an example to solve the problem of group decision-making of multi attributes. Table 1 will give the neutrosophic linguistic terms “Very Small” (VS), “Small” (S), “Intermediate Small” (IS), “Intermediate” (I), “Intermediate Large” (IL), “Large” (L), “Very Large.”

**Table 1:** Neutrosophic triangular membership value.

Very small (VS)	$$\left(\begin{array}{c}\left(\left(\mathrm{0,0},0\right),\left(\mathrm{0,0},0\right),\left(\mathrm{0,0},0\right),\left(\mathrm{0.07,0.02,0.01}\right),\left(\mathrm{0.7,0.2,0.1}\right),\left(\mathrm{0.7,0.2,0.1}\right)\right),\\ ((\mathrm{0,0},0),(\mathrm{0,0},0),(\mathrm{0,0},0),(\mathrm{0.035,0.01,0.005}),(\mathrm{0.63,0.18,0.09}),(\mathrm{0.63,0.18,0.09}))\end{array}\right)$$
Small (S)	$$\left(\begin{array}{c}\left(\left(\mathrm{0,0},0\right),\left(\mathrm{0.07,0.02,0.01}\right),\left(\mathrm{0.07,0.02,0.01}\right),\left(\mathrm{0.21,0.06,0.03}\right),\left(\mathrm{0.7,0.2,0.1}\right),\left(\mathrm{0.7,0.2,0.1}\right)\right),\\ \left((\mathrm{0.035,0.01,0.005}),(\mathrm{0.07,0.02,0.01}),(\mathrm{0.07,0.02,0.01}),(\mathrm{0.14,0.04,0.02}),(\mathrm{0.63,0.18,0.09}),(\mathrm{0.63,0.18,0.09})\right)\end{array}\right)$$
Intermediate small (IS)	$$\left(\begin{array}{c}\left(\left(\mathrm{0.07,0.02,0.01}\right),\left(\mathrm{0.21,0.06,0.03}\right),\left(\mathrm{0.21,0.06,0.03}\right),\left(\mathrm{0.35,0.1,0.05}\right),\left(\mathrm{0.7,0.2,0.1}\right),\left(\mathrm{0.7,0.2,0.1}\right)\right),\\ ((\mathrm{0.14,0.04,0.02}),(\mathrm{0.21,0.06,0.03}),(\mathrm{0.21,0.06,0.03}),(\mathrm{0.28,0.08,0.04}),(\mathrm{0.63,0.18,0.09}),(\mathrm{0.63,0.18,0.09}))\end{array}\right)$$
Intermediate (L)	$$\left(\begin{array}{c}\left(\left(\mathrm{0.21,0.06,0.03}\right),\left(\mathrm{0.35,0.1,0.05}\right),\left(\mathrm{0.35,0.1,0.05}\right),\left(\mathrm{0.49,0.14,0.07}\right),\left(\mathrm{0.7,0.2,0.1}\right),\left(\mathrm{0.7,0.2,0.1}\right)\right),\\ \left((\mathrm{0.28,0.08,0.04}),(\mathrm{0.35,0.1},0.05),(\mathrm{0.35,0.1,0.05}),(\mathrm{0.42,0.12,0.06}),(\mathrm{0.63,0.18,0.09}),(\mathrm{0.63,0.18,0.09})\right)\end{array}\right)$$
Intermediate large (IL)	$$\left(\begin{array}{c}\left(\left(\mathrm{0.35,0.1,0.05}\right),\left(\mathrm{0.49,0.14,0.07}\right),\left(\mathrm{0.49,0.14,0.07}\right),\left(\mathrm{0.63,0.18,0.09}\right),\left(\mathrm{0.7,0.2,0.1}\right),\left(\mathrm{0.7,0.2,0.1}\right)\right),\\ ((\mathrm{0.42,0.12,0.06}),(\mathrm{0.49,0.14,0.07}),(\mathrm{0.49,0.14,0.07}),(\mathrm{0.56,0.16,0.08}),(0.63,\mathrm{0.18,0.09}),(\mathrm{0.63,0.18,0.09}))\end{array}\right)$$
Large (L)	$$\left(\begin{array}{c}\left(\left(\mathrm{0.49,0.14,0.07}\right),\left(\mathrm{0.63,0.18,0.09}\right),\left(\mathrm{0.63,0.18,0.09}\right),\left(\mathrm{0.7,0.2,0.1}\right),\left(\mathrm{0.7,0.2,0.1}\right),\left(\mathrm{0.7,0.2,0.1}\right)\right),\\ ((\mathrm{0.56,0.16,0.08}),(\mathrm{0.63,0.18,0.09}),(\mathrm{0.63,0.18,0.09}),(\mathrm{0.665,0.19,0.095}),(\mathrm{0.63,0.18,0.09}),(\mathrm{0.63,0.18,0.09}))\end{array}\right)$$
Very large (VL)	$$\left(\begin{array}{c}\left(\left(\mathrm{0.63,0}.\mathrm{18,0.09}\right),\left(\mathrm{0.7,0.2,0.1}\right),\left(\mathrm{0.7,0.2,0.1}\right),\left(\mathrm{0.7,0.2,0.1}\right),\left(\mathrm{0.7,0.2,0.1}\right),\left(\mathrm{0.7,0.2,0.1}\right)\right),\\ ((\mathrm{0.665,0.19,0.095}),(\mathrm{0.7,0.2,0.1}),(\mathrm{0.7,0.2,0.1}),(\mathrm{0.7,0.2,0.1}),(\mathrm{0.63,0.18,0.09}),(\mathrm{0.63,0.18,0.09}))\end{array}\right)$$
  Here the problem deals with the manufacturing company, which is searching for the best supplier for assembling the critical parts. In this problem, there are three suppliers ${y}_{1},{y}_{2},{y}_{3}$ with the four attributes (${g}_{1}:$ product quality, ${g}_{2}:$ factors risk, ${g}_{3}:$ Supplier service performance, ${g}_{4}:$ profile of the supplier) whose corresponding neutrosophic weight vector is given by $\left(T{w}_{i},I{w}_{i},F{w}_{i}\right)=\left(((\mathrm{0.21,0.06,0.03}),(\mathrm{0.105,0.03,0.015}),(\mathrm{0.14,0.04,0.02}),(\mathrm{0.245,0.07,0.035}))\right)$. A group is formed with expert members, with three members say ${D}_{1},{D}_{2}$ and ${D}_{3}$ an weight vectors $\left(T{\lambda }_{i},I{\lambda }_{i},F{\lambda }_{i}\right)=\left((\mathrm{0.21,0.06,0.03}),(\mathrm{0.315,0.09,0.045}),(\mathrm{0.175,0.05,0.025})\right)$ in each decision area. With the help of the different attributes ${g}_{i}, (i=\mathrm{1,2},\mathrm{3,4})$ to the global suppliers ${y}_{1, }{y}_{2},{y}_{3}$ potential, the uses of linguistic terms by the experts ${D}_{1},{D}_{2}$ and ${D}_{3}$ is shown in Table 2.

**Table 2:** Linguistic terms of the decision makers.

Attributes	Product quality $\left({g}_{1}\right)$			Factors risk $\left({g}_{2}\right)$			Supplier service performance $\left({g}_{3}\right)$			Profile of the supplier $\left({g}_{4}\right)$		
Alternatives	${y}_{1}$	${y}_{2}$	${y}_{3}$	${y}_{1}$	${y}_{2}$	${y}_{3}$	${y}_{1}$	${y}_{2}$	${y}_{3}$	${y}_{1}$	${y}_{2}$	${y}_{3}$
Decision maker ${D}_{1}$	IL	L	VL	L	IL	VL	VL	L	I	VL	L	L
Decision Maker ${D}_{2}$	L	IL	L	VL	L	VL	L	VL	IL	L	VL	VL
Decision maker ${D}_{3}$	IL	L	IL	L	VL	L	L	VL	IL	L	L	VL
  Here all the attributes are considered to benefit attributes except factors risk $\left({g}_{2}\right)$ by using Eq. (14) and Tables 2 and 3 is obtained by normalizing it.

**Table 3:** Linguistic terms of the decision makers after normalized.

Attributes	Product quality $\left({g}_{1}\right)$			Factors risk $\left({g}_{2}\right)$			Supplier service performance $\left({g}_{3}\right)$			Profile of the supplier $\left({g}_{4}\right)$		
Alternatives	${y}_{1}$	${y}_{2}$	${y}_{3}$	${y}_{1}$	${y}_{2}$	${y}_{3}$	${y}_{1}$	${y}_{2}$	${y}_{3}$	${y}_{1}$	${y}_{2}$	${y}_{3}$
Decision maker ${D}_{1}$	IL	L	VL	S	IS	VS	VL	L	I	VL	L	L
Decision maker ${D}_{2}$	L	IL	L	VS	S	VS	L	VL	IL	L	VL	VL
Decision maker ${D}_{3}$	IL	L	IL	S	VS	S	L	VL	IL	L	L	VL
  Using Table 1, the individual normalized neutrosophic members were aggregated into the normalized neutrosophic decision matrix $\left(T{\mathcal{R}}^{k},I{\mathcal{R}}^{k},F{\mathcal{R}}^{k}\right)={\left(T{A}_{ij}^{k},{IA}_{ij}^{k},F{A}_{ij}^{k}\right)}_{3X4}(k=\mathrm{1,2},3)$ into the collective neutrosophic matrices $\left(T\mathcal{R},I\mathcal{R},F\mathcal{R}\right)={\left(T{A}_{ij},{IA}_{ij},F{A}_{ij}\right)}_{3X4}$ which is given as follows,
  $$\left(T\mathcal{R},I\mathcal{R},F\mathcal{R}\right)=\left(\begin{array}{ccc}\left(T{A}_{11},{IA}_{11},F{A}_{11}\right)& \left(T{A}_{12},{IA}_{12},F{A}_{12}\right)& \begin{array}{cc}\left(T{A}_{13},{IA}_{13},F{A}_{13}\right)& \left(T{A}_{14},{IA}_{14},F{A}_{14}\right)\end{array}\\ \left(T{A}_{21},{IA}_{21},F{A}_{21}\right)& \left(T{A}_{22},{IA}_{22},F{A}_{22}\right)& \begin{array}{cc}\left(T{A}_{23},{IA}_{23},F{A}_{23}\right)& \left(T{A}_{24},{IA}_{24},F{A}_{24}\right)\end{array}\\ \left(T{A}_{31},{IA}_{31},F{A}_{31}\right)& \left(T{A}_{32},{IA}_{32},F{A}_{32}\right)& \begin{array}{cc}\left(T{A}_{33},{IA}_{33},F{A}_{33}\right)& \left(T{A}_{34},{IA}_{34},F{A}_{34}\right)\end{array}\end{array}\right)$$
Step 2: By taking $s=t=1$. Using Eq. (14), the neutrosophic weighted Bonferroni mean is given by the following value:
  $$\left(T{d}_{1},I{d}_{1},F{d}_{1}\right)=\left(\begin{array}{c}\left(\left(\mathrm{0.86,0.75,0.73}\right),\left(\mathrm{1.17,1.05,1.03}\right),\left(\mathrm{1.17,1.05,1.03}\right),\left(\mathrm{1.19,1.06,1.03}\right),\left(\mathrm{1.2,1.06,1.03}\right)\left(\mathrm{1.2,1.06,1.03}\right)\right),\\ \left((\mathrm{1.16,1.05,1.03}),(\mathrm{1.17,1.05,1.03}),(1.17,\mathrm{1.05,1.03}),(\mathrm{1.18,1.05,1.03}),(\mathrm{1.2,1.06,1.03})(\mathrm{1.2,1.06,1.03})\right)\end{array}\right)$$
  $$\left(T{d}_{2},I{d}_{2},F{d}_{2}\right)=\left(\begin{array}{c}\left(\left(\mathrm{1.14,1.05,1.03}\right),\left(\mathrm{1.17,1.05,1.03}\right),\left(\mathrm{1.17,1.05,1.03}\right),\left(\mathrm{1.19,1.06,1.03}\right),\left(\mathrm{1.2,1.06,1.03}\right)\left(\mathrm{1.2,1.06,1.03}\right)\right),\\ \left((\mathrm{1.16,1.05,1.03}),(\mathrm{1.17,1.05,1.03}),(\mathrm{1.17,1.05,1.03}),(\mathrm{1.19,1.06,1.03}),(\mathrm{1.2,1.06,1.03})(\mathrm{1.2,1.06,1.03})\right)\end{array}\right)$$
  $$\left(T{d}_{3},I{d}_{3},F{d}_{3}\right)=\left(\begin{array}{c}\left(\left(\mathrm{0.85,0.75,0.73}\right),\left(\mathrm{1.15,1.05,1.03}\right),\left(\mathrm{1.15,1.05,1.03}\right),\left(\mathrm{1.19,1.05,1.03}\right),\left(\mathrm{1.2,1.06,1.03}\right)\left(\mathrm{1.2,1.06,1.03}\right)\right),\\ \left((\mathrm{1.15,1.05,1.03}),(\mathrm{1.15,1.05,1.03}),(\mathrm{1.15,1.05,1.03}),(\mathrm{1.18,1.05,1.03}),(\mathrm{1.2,1.06,1.03})(\mathrm{1.2,1.06,1.03})\right)\end{array}\right)$$
Step 3: By using Eqs. (3) and (4), neutrosophic lower and upper mean value is given as
  $$\left(T\widetilde{M}\left({d}_{1}\right),I\widetilde{M}\left({d}_{1}\right),F \widetilde{M}\left({d}_{1}\right)\right)=\left((\mathrm{0.440,0.422,0.377}),(\mathrm{0.222,0.197,0})\right)$$
  $$\left(T\widetilde{M}\left({d}_{2}\right),I\widetilde{M}\left({d}_{2}\right),F \widetilde{M}\left({d}_{2}\right)\right)=\left((\mathrm{0.441,0.422,0.377}),(\mathrm{0.222,0.197,0})\right)$$
  $$\left(T\widetilde{M}\left({d}_{3}\right),I\widetilde{M}\left({d}_{3}\right),F \widetilde{M}\left({d}_{3}\right)\right)=\left((\mathrm{0.444,0.417,0.375}),(\mathrm{0.215,0.195,0})\right)$$
Step 4: Then use (5) to determine the value of possibility neutrosophic degree is given as follows:
  $$\left(p\left({TN}_{1}\succcurlyeq {TN}_{1}\right),p\left({IN}_{1}\succcurlyeq {IN}_{1}\right),p\left(F{N}_{1}\succcurlyeq {FN}_{1}\right)\right)=\left(\mathrm{0.5,0.5,0.5}\right).$$
  $$\left(p\left({TN}_{1}\succcurlyeq {TN}_{2}\right),p\left({IN}_{1}\succcurlyeq {IN}_{2}\right),p\left(F{N}_{1}\succcurlyeq {FN}_{2}\right)\right)=(\mathrm{0.4987,0.5002,0.5})$$
  $$\left(p\left({TN}_{1}\succcurlyeq {TN}_{3}\right),p\left({IN}_{1}\succcurlyeq {IN}_{3}\right),p\left(F{N}_{1}\succcurlyeq {FN}_{3}\right)\right)=(\mathrm{0.5036,0.5083,0.5016})$$
  $$\left(p\left({TN}_{2}\succcurlyeq {TN}_{1}\right),p\left({IN}_{2}\succcurlyeq {IN}_{1}\right),p\left(F{N}_{2}\succcurlyeq {FN}_{1}\right)\right)=\left(\mathrm{0.5013,0.4998,0.49997}\right)$$
  $$\left(p\left({TN}_{2}\succcurlyeq {TN}_{2}\right),p\left({IN}_{2}\succcurlyeq {IN}_{2}\right),p\left(F{N}_{2}\succcurlyeq {FN}_{2}\right)\right)=(\mathrm{0.5,0.5,0.5})$$
  $$\left(p\left({TN}_{2}\succcurlyeq {TN}_{3}\right),p\left({IN}_{2}\succcurlyeq {IN}_{3}\right),p\left(F{N}_{2}\succcurlyeq {FN}_{3}\right)\right)=\left(\mathrm{0.5048,0.5081,0.5016}\right)$$
  $$\left(p\left({TN}_{3}\succcurlyeq {TN}_{1}\right),p\left({IN}_{3}\succcurlyeq {IN}_{1}\right),p\left(F{N}_{3}\succcurlyeq {FN}_{1}\right)\right)=(\mathrm{0.4964,0.4917,0.4984})$$
  $$\left(p\left({TN}_{3}\succcurlyeq {TN}_{2}\right),p\left({IN}_{3}\succcurlyeq {IN}_{2}\right),p\left(F{N}_{3}\succcurlyeq {FN}_{2}\right)\right)=(\mathrm{0.4952,0.4919,0.4984})$$
  $$\left(p\left({TN}_{3}\succcurlyeq {TN}_{3}\right),p\left({IN}_{3}\succcurlyeq {IN}_{3}\right),p\left(F{N}_{3}\succcurlyeq {FN}_{3}\right)\right)=(\mathrm{0.5,0.5,0.5})$$
Step 5: Then find the neutrosophic preference matrix $\left(TP,IP,FP\right)$ using (10) which is:
  $$\left(TP,IP,FP\right)=\left(\begin{array}{ccc}(\mathrm{0.5,0.5,0.5})& (\mathrm{0.4987,0.5002,0.5})& (\mathrm{0.5036,0.5083,0.5016})\\ (\mathrm{0.5013,0.4998,0.49997})& (\mathrm{0.5,0.5,0.5})& (\mathrm{0.5048,0.5081,0.5016})\\ (\mathrm{0.4964,0.4917,0.4984})& (\mathrm{0.4952,0.4919,0.4984})& (\mathrm{0.5,0.5,0.5})\end{array}\right)$$
Step 6: By using Eq. (11) the neutrosophic ranking value $\mathcal{R}\left(T{d}_{i},I{d}_{i},F{d}_{i}\right)$ is:
  $$\mathcal{R}\left(T{d}_{1},I{d}_{1},F{d}_{1}\right)=\left(\mathrm{1.1227,1.1233,1.1226}\right)$$
  $$\mathcal{R}\left(T{d}_{2},I{d}_{2},F{d}_{2}\right)=(\mathrm{1.1230,1.1232,1.1226})$$
  $$\mathcal{R}\left(T{d}_{3},I{d}_{3},F{d}_{3}\right)=(\mathrm{1.1217,1.1209,1.1222})$$
Step 7: Finally using (2), the rank of the alternatives $\mathcal{R}({d}_{i})$ are obtained as
  $$\mathcal{R}\left({d}_{1}\right)=5.5517,\mathcal{R}\left({d}_{2}\right)=5.5533,\mathcal{R}\left({d}_{3}\right)=5.5481.$$
Step 8: The ranking will be given in the descending order $\mathcal{R}\left({d}_{2}\right)=5.5533>\mathcal{R}\left({d}_{1}\right)=5.5517>\mathcal{R}\left({d}_{3}\right)=5.5481$, therefore, the order will be given to ${y}_{2}$ has first preference. For this the proposed method does not have any complication in the calculation of identifying the best supplier.
Step 9: Now we will give a comparative study with the previous methods in Table 4. Even though the ranking order is the same as the previous methods, the proposed method will overcome the drawbacks of the previous methods. For the same problem, method 1 has the disadvantage of calculating the upper and lower value of the preference matrix, but in the proposed method, it was calculated simultaneously. Similarly, the possibility degree is also calculated twice in method 1, whereas it was a single calculation in the proposed method. In method 2, the computation of the possibility degree value is complicated, whereas, in the proposed method, it is simpler to calculate. Here TITRWAA operator is used in method 2, which is comparatively lengthier than the proposed method. Hence it is better than the previous method. In method 3, it failed to apply to other types of decision-making problems, such as choice in investment making, problems without the information of the weights of the attributes the decision-makers will provide. However, we can apply the neutrosophic formula to the above situations in the proposed method.

**Table 4:** Comparison with previous methods.

No	Methods	Result	Conclusion
1	Chen and Lee^20^	$\mathcal{R}\left({d}_{1}\right)=0.6212,\mathcal{R}\left({d}_{2}\right)=0.8383, \mathcal{R}\left({d}_{3}\right)=0.5405$	$\mathcal{R}\left({d}_{2}\right)>R\left({d}_{1}\right)>R\left({d}_{3}\right)$
2	Hu et al.^25^	$\mathcal{R}\left({d}_{1}\right)=0.3250,\mathcal{R}\left({d}_{2}\right)=0.4036, \mathcal{R}\left({d}_{3}\right)=0.2715$	$\mathcal{R}\left({d}_{2}\right)>R\left({d}_{1}\right)>R\left({d}_{3}\right)$
3	Gong et al.^27^	$\mathcal{R}\left({d}_{1}\right)=0.2887,\mathcal{R}\left({d}_{2}\right)=0.4896, \mathcal{R}\left({d}_{3}\right)=0.2218$	$\mathcal{R}\left({d}_{2}\right)>R\left({d}_{1}\right)>R\left({d}_{3}\right)$
4	Proposed method	$\mathcal{R}\left({d}_{1}\right)=5.5517,\mathcal{R}\left({d}_{2}\right)=5.5533, \mathcal{R}\left({d}_{3}\right)=5.5481$	$\mathcal{R}\left({d}_{2}\right)>R\left({d}_{1}\right)>R\left({d}_{3}\right)$

## Conclusion

The conventional Bonferroni mean operator and possibility degree have been extended to the trapezoidal and triangular neutrosophic environment to better organize and model the uncertainties and indeterminacy inside multi-attribute decision analysis. In FMAGDM, the neutrosophic Bonferroni operator can aggregate individual preferences or evaluations of multiple decision-makers. Neutrosophic environments, as opposed to trapezoidal and triangular ones, can convey the decision-makers' uncertainty, indeterminacy, and ambiguity. We have introduced a novel approach for NMAGDM based on the neutrosophic possibility degree and the TITRNWBM operator. We also offer numerical examples to show how the proposed method's NMAGDM process works for a manufacturing company searching for the best supplier for assembling the critical parts. The outcome demonstrates that the suggested approach gives us a practical means of resolving NMAGDM issues based on trapezoidal and triangular neutrosophic environments. Future work will apply the proposed techniques to other decision-making issues involving Plithogenic environments.

## Data Availability

All data generated or analysed during this study are included in this published article.

## Abbreviations

NMAGDM: Neutrosophic Multi-Attribute Group Decision Making
DMs: Decision makers
TITENBM: Trapezoidal and triangular neutrosophic Bonferroni mean
TITRNWBM: Trapezoidal and triangular neutrosophic weighted Bonferroni mean
FMAGDM: Fuzzy multi-attributes group decision-making
TITRWAA: Trapezoidal and triangular neutrosophic weighted arithmetic
TOPSIS: The Technique for Order of Preference by Similarity to Ideal Solution
